# Physical and chemical mechanisms in oxide-based resistance random access memory

**DOI:** 10.1186/s11671-015-0740-7

**Published:** 2015-03-12

**Authors:** Kuan-Chang Chang, Ting-Chang Chang, Tsung-Ming Tsai, Rui Zhang, Ya-Chi Hung, Yong-En Syu, Yao-Feng Chang, Min-Chen Chen, Tian-Jian Chu, Hsin-Lu Chen, Chih-Hung Pan, Chih-Cheng Shih, Jin-Cheng Zheng, Simon M Sze

**Affiliations:** Department of Materials and Optoelectronic Science, National Sun Yat-Sen University, Kaohsiung, Taiwan; Department of Physics, National Sun Yat-Sen University, Kaohsiung, Taiwan; Information Technology Center, BGP, China National Petroleum Corporation, Beijing, China; Microelectronics Research Center, The University of Texas at Austin, Austin, TX 78758 USA; Department of Mechanical and Electro-Mechanical Engineering, National Sun Yat-Sen University, Kaohsiung, Taiwan; Department of Physics, Xiamen University, Xiamen, 361005 China; Department of Electrical Engineering, Stanford University, Stanford, CA 94305 USA

**Keywords:** RRAM, Silicon oxide, Graphene oxide, Physical mechanism, Supercritical fluids

## Abstract

In this review, we provide an overview of our work in resistive switching mechanisms on oxide-based resistance random access memory (RRAM) devices. Based on the investigation of physical and chemical mechanisms, we focus on its materials, device structures, and treatment methods so as to provide an in-depth perspective of state-of-the-art oxide-based RRAM. The critical voltage and constant reaction energy properties were found, which can be used to prospectively modulate voltage and operation time to control RRAM device working performance and forecast material composition. The quantized switching phenomena in RRAM devices were demonstrated at ultra-cryogenic temperature (4K), which is attributed to the atomic-level reaction in metallic filament. In the aspect of chemical mechanisms, we use the Coulomb Faraday theorem to investigate the chemical reaction equations of RRAM for the first time. We can clearly observe that the first-order reaction series is the basis for chemical reaction during reset process in the study. Furthermore, the activation energy of chemical reactions can be extracted by changing temperature during the reset process, from which the oxygen ion reaction process can be found in the RRAM device. As for its materials, silicon oxide is compatible to semiconductor fabrication lines. It is especially promising for the silicon oxide-doped metal technology to be introduced into the industry. Based on that, double-ended graphene oxide-doped silicon oxide based via-structure RRAM with filament self-aligning formation, and self-current limiting operation ability is demonstrated. The outstanding device characteristics are attributed to the oxidation and reduction of graphene oxide flakes formed during the sputter process. Besides, we have also adopted a new concept of supercritical CO_2_ fluid treatment to efficiently reduce the operation current of RRAM devices for portable electronic applications.

## Review

### Introduction

If there was no record of culture relics and words for civilization, the era never existed in the past. People portrayed everything of life at different times in natural materials, such as stone, metal, and wood, to inherit the experience of life and prove the existence of once to be passed along. However, because the carriers of civilizations are bulky and difficult to be depicted and wrote by people, the life experience and scientific civilization can only be spread in the same place. When a major disaster or a big famine comes to here, people are forced to migrate to other environments, leading to the accumulated experience and knowledge of civilization making a fresh start. With the evolution of the times, the inventions of paper and printing bring people a convenient and light carrier of words, which causes the appearance of book. Not only the knowledge and ideas can be duplicated in books, but also they can be easily spread around the world. These inventions will make many people share the achievements of civilization. Owing to the continuous accumulation and growth of knowledge, the amount of papers and books used to store all civilizations also increase dramatically. In order to conserve these books and record the civilizations, people continue to set up the library around the world. But the space is limited; people are bound to find new ways to store knowledge. As the advent of the digital age, the problem of expansion of knowledge and information will be solved for human being.

Since the advent of the digital era, the way of digital memory is continuously in progress. Since the holes cards, magnetic matrix, tapes, magnetic cards, and hard disk used successively to nowadays flash memory invented by Simon M. Sze, the capacity and density of digital memory continues to upgrade and the speed also continuously increases. With the rise of portable electronic and Internet operations, knowledge of people around the world communicates and delivers fast anytime and anywhere. The uploaded content is no longer just text but using the manner of photos, video, and sound to record moments of life in detail, even using the websites, blogs, Facebook, etc. to communicate with each other. These progress and development are attributed to digital electronic technology and digital memory evolution.

Investigating how to enhance the progress of memory is the responsibility and obligations of people in every age. The more detailed heritage of civilization is achieved, the better the performance memory will be required. Resistance random access memory (RRAM) is a good direction of future development in memory. In recent years, continuous improvement and in-depth investigation in both materials and electrical switching mechanism not only make a breakthrough in performance of digital non-volatile memory but also look for other possibility of memory functionality.

With the demand of multi-functional electronic devices increasing greatly in the world, the investigation of device physics will be important for IC industry [1]. Furthermore, the multi-functional portable electronic products should integrate with memory [2-24], display [25-49], logic devices [50-57], and functional devices (e.g. sensor) [58,59] according to device physics and fabrication technology. Among the above-mentioned devices, the memory is important for the cultural evolution of human being. Since the origin of human civilization, different ways and media were used in transmitting historic experience and recording development of technology. From the earliest cuneiform scripts and pictograph to Greek and Latin and eventually to modern digital signal, ways to record information have been transformed from primitive stone slab and metalware carving to the Chinese invention of papermaking and printing and finally developed into nowadays memory devices [60,61]. With the transition of recording media becoming more portable and most importantly cheaper, it has promoted the development and dissemination of human culture. In addition, owing to the flourishing IC industry and notably the invention of flash non-volatile memory by Simon M. Sze [62], technology that records and transmits information has been particularly changed and boosted, laying a solid foundation for the extensive use and wide spreading of portable electronic products such as iPods, digital cameras, smart TVs, and smartphones. However, flash memory cannot keep pace with the sustained reduction of IC gate-length dimension and meanwhile fulfilling the storage needs due to the physical limit of gate oxide. Among emerging memory technologies [63-68], RRAM has the potential to become the ultimate next-generation non-volatile memory for its attributes of simple structure, good performance, and scalability [69-109]. Various materials have been reported owning RRAM properties including the extensively investigated grapheme [110-112] and its based materials. Also, graphene-based broken flakes materials are the most promising candidates to be applied in the industry owing to its special properties and relative easiness to fabricate [112].

**Figure 1:**
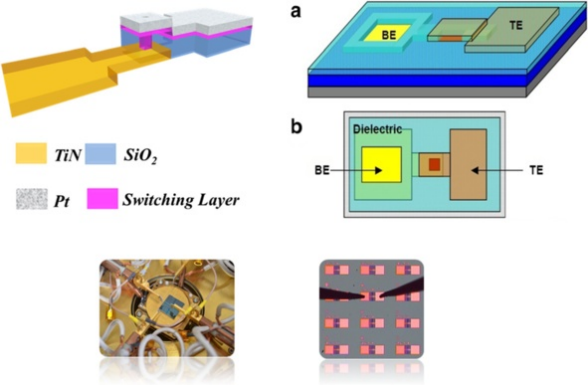
**3D device structure and fabrication process of patterned substrate. a, b** are the device whole view and top view, respectively. The electrical measurement of the devices was conducted in a variable-temperature microprobe system.

In this review, we provide an overview of our work in resistive switching mechanisms on oxide-based RRAM devices in recent years. The schematic structure of RRAM devices is shown in the top panel of Figure 1. The electrical measurement of the oxide-based RRAM devices was conducted in a variable temperature (4 to 400 K) microprobe system as shown in the bottom of Figure 1. Based on the investigation of physical mechanisms, we comprehensively research the influences and working characteristics for RRAM devices with different materials, device structures, and treatment methods.

## Physical mechanisms in oxide-based RRAM devices

Recently, the switching mechanisms in metal oxide RRAM still existed various controversies. One of the most reasonable physical mechanisms is the filament formation and rupture. Many previous reports focused on the discussion of characteristics factors of filament in low-resistance state (LRS) and high-resistance state (HRS) such as resistance, temperature, and voltage [113-117]. However, there are few reports to discuss the physical mechanisms of dynamic set and reset processes in metal oxide RRAM. Therefore, we investigated the physical mechanisms of set and reset process by hafnium oxide RRAM devices.

### Physical mechanism of set process in hafnium oxide RRAM devices

In this study, set process was mainly investigated to explore the relationship between voltage and pulse rising time for oxide-based RRAM devices with Ti/HfO_x_/TiN structure. It demonstrates that formation of filaments was directly related to the total energy of applied power.

**Figure 2:**
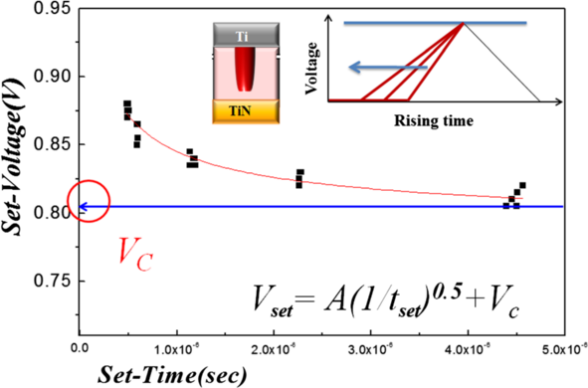
**Relationship of set-voltage versus set-time with different pulse rising time in the set process.**

In general, the set voltage for each specified RRAM device should be a constant, and the operating voltage needs to exceed the critical value so as to switch the device from HRS to LRS [113]. This study imposes a triangular wave under various leading time conditions with a fixed top voltage of 2 V and a trailing time of 100 ns. Same reset process was operated in DC mode to confirm the resistance at the same HRS before each setting process triggered by different leading time conditions. The critical set voltage (*V*_*C*_) of RRAM device, which is the minimal voltage for a filament path formation irrelative to time, can be obtained from various triangular rising time shown in Figure 2 through a specific designed circuit measurement by a pulse generator and an oscilloscope.

**Figure 3:**
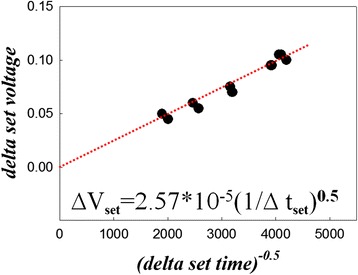
**Relationship of delta set voltage versus the delta set time with different pulse rising times in the setting process.**

Furthermore, the delta set voltage (*ΔV*_set_) can be defined as the difference between set voltage and critical set voltage from oscilloscope. The delta set time (*Δt*_set_) is defined as the time difference of corresponding set and critical set voltage. Figure 3 shows the experimental results of *ΔV*_set_ − *Δt*_set_ at different triangular rising times, which demonstrates that the setting process can be conformed by following formula through curve fitting:

1 $$ \varDelta {V}_{\mathrm{set}}=a{\left(\varDelta {t}_{\mathrm{set}}\right)}^{-0.5}=2.57\times {10^{-}}^5{\left(\varDelta {t}_{\mathrm{set}}\right)}^{\hbox{-} 0.5} $$

Therefore,

2 $$ {\left(\varDelta {V}_{\mathrm{set}}\right)}^2\times \varDelta {t}_{\mathrm{set}}=\mathrm{constant}. $$

If the resistive switching of metal oxide RRAM results from redox reaction mechanism, the reaction can only be triggered by sufficient energy based on electric chemical reaction theory. Therefore, we calculate the setting energy (*W*_RRAM_) over the critical voltage in the RRAM device from a critical position to a setting point as follows:

3 $$ \begin{array}{c}\hfill {W}_{\mathrm{RRAM}}={\displaystyle \underset{0}{\overset{\varDelta {t}_{\mathrm{set}}}{\int }}}\left(\varDelta {I}_{\mathrm{RRAM}}\times \varDelta {V}_{\mathrm{RRAM}}\right)\cdot \mathrm{d}\mathrm{t}\hfill \\ {}\hfill =\left[{\left(\varDelta {V}_{\mathrm{set}}\right)}^2\times \varDelta {t}_{\mathrm{set}}\right]\;\left(1/3{R}_{\mathrm{RRAM}}\right)\hfill \end{array} $$

The setting energy of RRAM is constant in the same resistance state, which is proved by substituting Equation 2 into 3. From the discussion above, we present that resistive switching is directly related to a critical switching energy, which has a powerful influence on the redox reaction of RRAM set process. We think that the critical switching energy is the energy to interrupt the oxide chemical bond in the metal oxide RRAM devices during redox reaction of set process.

### Atomic-level quantized reaction in RRAM reset process

From the study in previous literatures, the procedure of resistive switching is dominated by a sequential redox reaction [68,114,118-120]. The redox reaction will cause the formation and rupture of filament, leading to the resistive switching between HRS and LRS. However, the atoms involved in the reaction procedure are so few that we need to carefully design an experiment to verify the reset phenomenon in RRAM with Ti/HfO_x_/TiN structure. In order to examine the reset phenomenon of RRAM due to atomic-level reaction, the current versus voltage (*I-V*) would be analyzed at the ultra-cryogenic temperature of 4 K.

**Figure 4:**
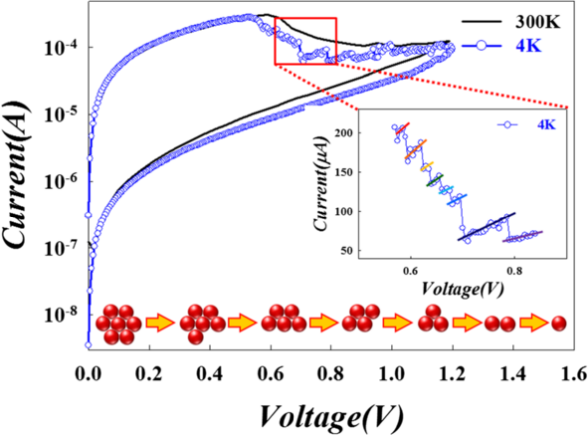
**Quantization reaction for a RRAM at ultra-cryogenic temperature (4K).** The inset diagram in the bottom part is an illustration to expound the discontinuous resistance variation due to quantized atomic reaction during reset process [5].

The continuous *I-V* curve of the RRAM device in the room temperature can be observed by the black line in Figure 4. In contrast, a special discontinuous trend of *I-V* curve was found at 4 K. The regularity at some regime of *I-V* curve can be observed by the ratio of voltage and current. Based on the Ohm’s law of *R* = *V*/*I*, the different ratios of voltage and current represent different resistance values. The resistance values revealed a regular phenomenon of decreased variation step by step with reset reaction procedure shown in the magnified inset of Figure 4. This quantization reaction is a different physical phenomenon with the conductance quantization in nanoscale conductive filament [121,122].

**Figure 5:**
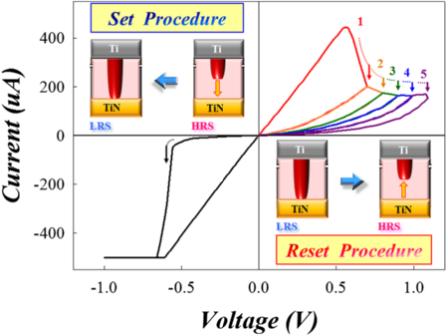
**Abrupt set procedure and multistep reset procedure for oxide-based RRAM device** **[** 5 **].**

The typical resistance switching characteristics of Ti/HfO_x_/TiN cells were measured by DC voltage sweep mode shown in Figure 5. For the operation of RRAM, an irreversible forming procedure is required to activate the as-fabricated RRAM cells. A compliance current of 500 μA was set to prevent permanent breakdown during RRAM operation in DC-voltage sweeping mode. A sudden increase of the current was observed at the voltage of about 3.5 V to achieve the forming procedure. After forming process, a gradual descent of current interpreted the cell that was switched back to HRS from LRS while a positive bias was swept over the reset voltage (*V*_reset_, 0.55 V), which is called as ‘reset procedure’ and due to the rupture of the filament. Conversely, as the negative bias was swept over the set voltage (*V*_set_, −0.55 V), the RRAM cell will switch from HRS to LRS, i.e. ‘set procedure,’ which is attributed to the formation of filament. The inset diagram in the upper left shows that the set procedure is the transformation of HRS changed to LRS. The inset diagram in the lower right shows the reset procedure, which can be divided into different resistive stages by altering the negative stop voltage of DC sweeping cycle (*V*_stop_). The resistance value increases with increasing the *V*_stop_, leading to the multi-level resistance state obtained by controlling the *V*_stop_.

**Figure 6:**
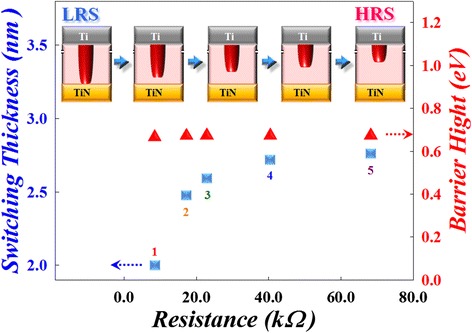
**Relationship among switching thickness, barrier height, and device resistance in the reset process** **[** 5 **].**

To investigate the switching mechanism, the multiple *I-V* curves in Figure 5 are fitted to analyze the carrier transport of switching layer. Firstly, a good linear relationship with a slope of 1 was found in the curve of ln(*I*) versus ln(*V*), indicating that the carrier transport in LRS is dominated by Ohmic conduction. Furthermore, the different states of reset *I-V* curves which represent the multi-high-resistance state (multi-HRS) from stage 1 to stage 5 in the Figure 5 were fitted to analyze their carrier transport mechanism. We found that the relationship in the curve of ln(*I*/*T*^2^) versus the square root of the applied voltage (*V*^1/2^) is linear. This demonstrated that Schottky emission is considered as the main transport mechanism in multi-HRS during the reset procedure. The major leakage current is contributed from the electrons crossing the potential energy barrier between the interface of switching layer and TiN electrode by the thermionic effect. According to the formula of Schottky emission, $$ J={A}^{**}{T}^2 exp\Big[\frac{-q\left({\varphi}_B-\sqrt{\raisebox{1ex}{$qV$}\!\left/ \!\raisebox{-1ex}{$4\pi {\varepsilon}_i{d}_{\mathrm{sw}}$}\right.}\right)}{kT} $$, where A** is the Richardson constant, the effective switching thickness (*d*sw) and energy barrier height (*φ*_*B*_) can be obtained from the slope and intercept of the plot of $$ \sqrt{V}- \ln \left(\frac{I}{T^2}\right) $$, respectively. The *ε*_*i*_ = *kε*_0_, and the *k* of HfO_2_ is about 25. Furthermore, the *d*sw and *φ*_*B*_ of different multi-HRS in Figure 5 can also be obtained through calculation. Its *d*sw and *φ*_*B*_ versus corresponding resistance for different multi-HRS in Figure 5 are shown in Figure 6. The results exhibit that the *φ*_*B*_ was fixed about 0.7 eV and independent with corresponding resistance in different multi-HRS. This phenomenon implicates that the switching layer in different multi-HRS showed similar material properties. However, the *d*sw was increased with the increase of resistance value in different multi-HRS during the reset procedure. The results are attributed to the continuous conduction filament that will be ruptured gradually with the increasing of *V*_stop_, as shown in the insets of upper site of Figure 6.

**Figure 7:**
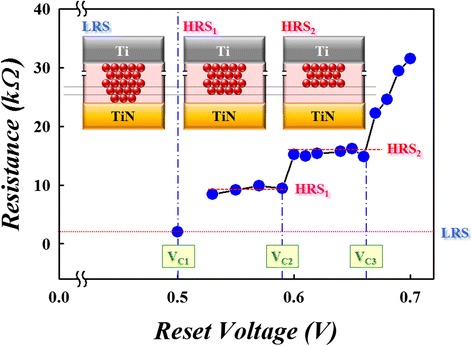
**Relationship of device resistance versus reset voltage at 77 K.** The reset voltages are operated within a fixed voltage range (0.5 V to 0.7 V) with 0.01 V interval for 1,000 s at 77 K to assure the reaction is accomplished completely.

In view of the analytic results for multi-HRS during reset procedure, the carrier transport mechanism is understood to be converted from Ohmic conduction to Schottky emission. This suggests that the conductive filament in initial LRS was oxidized through layer by layer by oxygen ions returned from the TiN electrode, and the filament was gradually disconnected from the TiN electrode to form a weak Schottky barrier during reset procedure. In the study, the experiment was designed to prove the oxidation procedure in conductive filament, which was observed by quantized resistance states with various reset voltage as shown in Figure 7. Each reset voltage was applied on device for a long time (1,000 s) to ensure complete oxidation of the conductive filament. Besides, the temperature of measurement environment was cooled down to 77 K to lessen thermodynamic effect during the reset procedure of RRAM. Figure 7 shows the correlation of the equilibrium resistance states with various reset voltage operation. The voltage bias was applied on the RRAM with suitable reaction time to initiate the oxidation of filament. The resistance states of RRAM start to change as the applied voltage exceed the first critical reaction voltage (*V*_C1_). The resistance states of RRAM were maintained at the first level of resistive states (HRS_1_) and the second level of high resistive states (HRS_2_) while the applied voltage was switched from *V*_C1_ to the second critical reaction voltage (*V*_C2_); and it was quite the same for the resistance change process when *V*_C2_ switched to the third critical reaction voltage (*V*_C3_). The resistance states of RRAM were transferred into higher resistance step by step as the reset voltage was raised gradually. The quantized reaction procedures for the RRAM transferred from the LRS to the HRS are illustrated in the upper site insets schematically. Moreover, the thickness of reaction layer will grow gradually, and the conductive filament becomes further away from the TiN electrode layer by layer. A higher reset voltage is necessary to supply enough electric field for the chemical reaction. Therefore, the resistance states would be kept until the reset voltage reaches the critical reaction electric field to initiate the next layer reaction of conductive filament. In this experiment, we demonstrated that the reset procedure of RRAM device is a layer-by-layer oxidation process. Therefore, atomic-level quantized oxidation must exist between the different reaction layers during reset procedure. This phenomenon can be observed obviously through the quantized current variation in ultra-cryogenic temperature (4 K) environment, which is shown in Figure 4.

**Figure 8:**
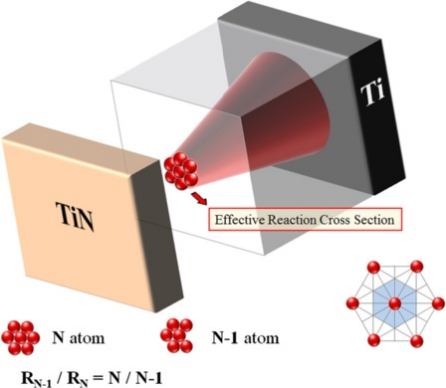
**Filament 3D speculation diagram.** The inset shows the hexagonal-close-packed structure, where the light blue region is the effective cross section area of each atom [5].

Based on the above experiment results, the reaction began from the first layer of conductive filament close to the TiN electrode during the reset procedure. During the reaction in the first layer, the effective cross section of filament will reduce and cause the variation of resistance in RRAM. Therefore, we propose a three-dimensional diagram of filament in the LRS of RRAM to represent the continuous conduction path between TiN and Ti electrodes as shown in Figure 8. The effective conduction area of the filament would be reduced due to oxygen atoms oxidized with filament one after another, leading to the rise of the resistance value of RRAM. Because the resistance value is inversely proportional to the effective conduction area determined by the number of component atoms of conduction filament, the ratio of resistance with and without *i* atoms removed away in effective cross section is *R*_*N* −_*i*/*R*_*N*_ = *N*/*N* − *i*. Therefore, the quantized variation of resistance can be attributed to the atomic-level reaction on the filament during the reset procedure. In order to clarify the switching mechanism, the resistance switching characteristics of RRAM were measured in the 4-K environment to eliminate the thermodynamic effects during reset procedure. The step-by-step resistance change shown in Figure 4 can be explained by the cross-sectional effective atom removal, which will result in the quantized reduction of the resistance.

**Figure 9:**
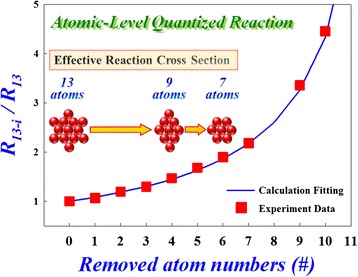
**Atomic-level quantized reset reaction resistance ratio.** The inset shows the cross-sectional numbers of effective atoms after reaction [5].

The ratio of initial and post-reactive numbers of atom corresponds to a specific ratio of resistance states during the reset procedure. Every transition resistance states can be extracted from the slopes of experimental data in Figure 4 for each quantized step of *I-V* curve at ultra-cryogenic temperature (4 K). Based on the above discussion for resistance calculation, the theoretical resistance ratio of effective cross section between beginning and intermediate states can be calculated during the reset process. The experimental values complied exactly with the theoretical calculation data as shown in Figure 9. The results demonstrate that the reaction of reset procedure is an atomic-level reaction, leading to the decrease of the effective cross-sectional conduction area, which externally exhibits as the rising of resistance. The original number of atoms is 13, which is calculated from the experimental data in the intermediate region of reset procedure as shown in the enlarge inset of Figure 4. Because the magnitude of change in resistance value is not so pronounced at the beginning of reset procedure, leading to the number of atoms on effective conduction filament area in initial LRS, it is difficult to observe through Figure 4. However, we can obtain the atomic numbers of effective cross section in the initial LRS by the relationship of resistance value and conduction area of filament.

Because the resistance value of the filament is roughly inversely proportional to the area A, the number of component atoms in the effective cross section of filament is proportional to A. The number of component atoms in the initial LRS (#_LRS_) can be obtained from the resistance of 13 effective atoms (#_13_) acquired from the experimental data in Figure 4: #_LRS_ = *R*_13_ × #_13_/*R*_LRS_ = 2,760 × 13/1,920 = 18.68. If the atoms in the filament are configured with a hexagonal lattice structure, the effective area of each atom can be indicated by the blue region of inset in Figure 8. While the lattice constant is 0.32 nm, the effective area (*A*_#_) of each atom is 0.0887 (nm^2^). The effective area (*A*_LRS_) of the initial LRS can be calculated as *A*_LRS_ = #_LRS_ × *A*_#_ = 18.68 × 0.0887 = 1.66 (nm^2^). According to the simple Ohm’s law, the estimated resistivity *ρ* is 319 (nΩ-m), which is approximately the theoretical value of metal hafnium (*ρ*_Hf_ = 331 (nΩ-m)). Therefore, we consider that the conductive filament for the carrier transport in the LRS is constructed with the elemental Hf. This is a first time to verify that the filament is constructed by Hf element in HfO*x* RRAM through the discussion of physical mechanisms during reset process.

## Chemical mechanisms in oxide-based RRAM devices

The first-order rate law analysis of the electrochemical reaction in Ni:SiO_2_ thin film resistance random access memory (RRAM) devices was investigated and discussed for the mechanism of reset process. According to the relationship between the resistive switching properties and the reaction time, the reaction rate constant (*k*) of the first-order reaction equation in reset process of the Ni:SiO_2_ RRAM devices was calculated and defined. A special constant voltage sampling (CVS) method was introduced to testify the reaction rate constant, from which we obtained the same value of *k*, confirming the same electrochemical reaction mechanism.

### Introduction of first-order rate law analysis

**Figure 10:**
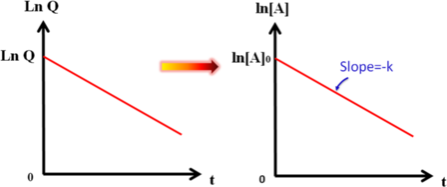
**The Ln(** ***Q*** **)-time and Ln(A)-time curves for first-order rate law analysis in RRAM device.**

To inspect the properties involved in the reset process, the proportional reaction rate and the reactant concentration of the first-order rate law in electrochemical reaction were used and discussed. The first-order rate law can be expressed as, in which [*A*], *t*, and *k* represent the reaction production concentration, reaction time and reaction rate constant. In addition, there also exists a relationship between the reaction production concentration [*A*] and the charge quantity *Q*, which can be expressed as [*Q*/(q*n)]/*V* = mole/*V* = [*A*]. In this equation, *Q*/(q*n) is defined as the mole of the reaction production as a whole, in which *n* is the number of the reaction and *q* is the charge carried by one-mole electrons. Besides, *V* is the volume of the resistive film, which can be viewed as a constant. From this equation, we find that the reaction production concentration [*A*] is proportional to the charge quantity *Q*. By substituting this equation into the first-order rate law equation, we can obtain that the reaction rate constant *k* directly correlates with the slope of In(*Q*)-time curve. Thus by drawing out the Ln(*Q*)-time curve, we can evaluate the variation of constant *k* as shown in Figure 10.

### Determination of chemical reaction rate for reset process in Ni:SiO_2_ RRAM

**Figure 11:**
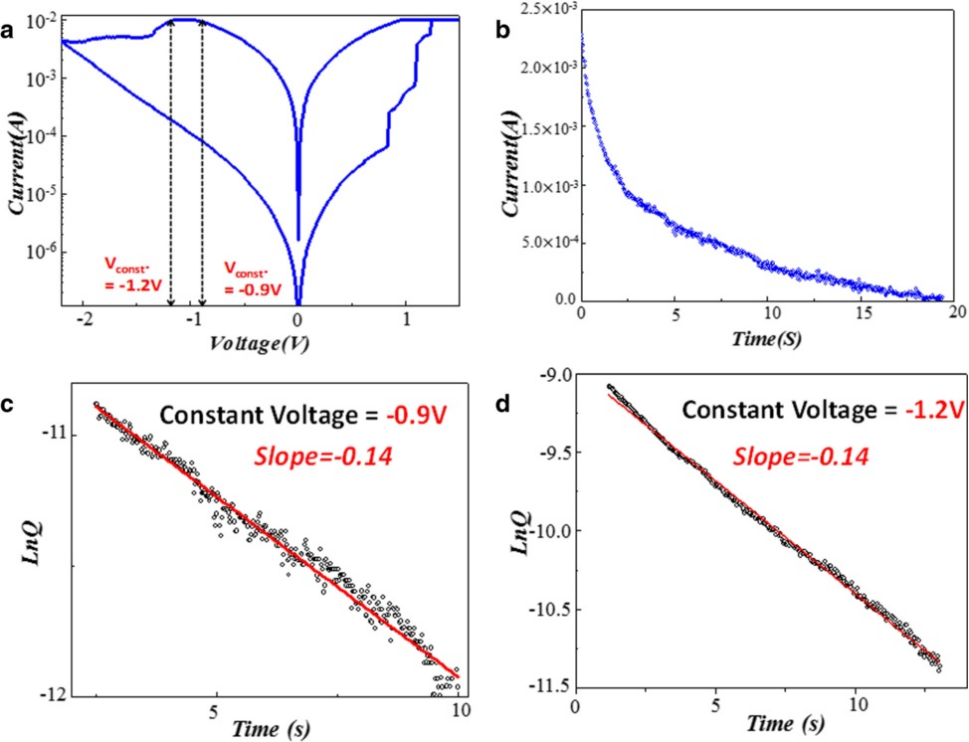
**Bipolar switching behavior, current-time sampling, and Ln(** ***Q*** **)-time fitting curves for −0.9 and −1.2 V. (a)** The typical bipolar switching behavior and the metal-insulator-metal (MIM) device structure and **(b)** the current-time sampling points of the Ni:SiO_2_ thin film RRAM device. **(c)** The Ln(*Q*)-time fitting curve for −0.9 V constant sampling voltage condition. **(d)** The Ln(*Q*)-time fitting curve for −1.2 V constant sampling voltage condition.

In this research, the reset voltage is defined as the point when obvious current drop exhibits and sometimes even accompanied with unstable *I-V* properties. Constant voltage sampling is an electrical measurement method, in which voltage bias sweeps from 0 V to the reset point and then remains unchanged. By keeping the voltage at the reset voltage, we sample the current for a period to obtain the continuing resistance change. The sampling voltage here works much more like a reading voltage, from which we can track the spontaneous chemical reaction triggered by the critical voltage point. And one constant voltage sampling result in the Ni:SiO_2_ RRAM is shown in Figure 11b. In this figure, the *y* and *x* axis represent current and time, respectively. From Figure 11b, we can see that the current still keeps decreasing even if the voltage remains at the reset point. Without increasing the operation voltage, continuous current drop manifests the lasting chemical reaction and this process sustains for more than 20 s. Figure 11c shows the first CVS results, and the sampling voltage is −0.9 V. By calculating *Q* from measurement results, we draw out the Ln(*Q*)-time curve, which can be seen in Figure 11c. With the increase of sampling time, Ln(*Q*) drops linearly within the first 10 s. From the red fitting curve, we find the slope of the Ln(*Q*)-time curve is −0.14. After the first CVS, another CVS measurement was conducted and the results are shown in Figure 11d. The reset voltage at this time is −1.2 V, but interestingly we get the same slope, which is −0.14. The second CVS lasts for 15 s. The same slope Ln(*Q*)-time curves implies the same reaction rate constant. It is quite common for RRAM to undergo slight current variation in the reset process owing to the stochastic oxidation reaction, but the same reaction rate constant involved in this random process unveils the similarities between relatively different reset situations. At the beginning of different reset processes, there will be a similar chemical reaction procedure in accordance with the first-order rate law and the reaction rate constant remains the same. Besides, even though the sampling voltages vary each other, the same *k* confirms the same reaction mechanism. This is quite different from our common view that higher voltage will lead to faster chemical reaction.

The current compliance was set with 10 mA and current-voltage curve is shown in Figure 11a. From the *I-V* curves, we can see the set voltage is around 1 V, and the reset voltage is about −1 V. Meanwhile, the set process is characterized by transient transformation, and the reset process is a gradual oxidation procedure, in which oxygen ions are propelled to rupture the conduction filament. Because of the random stochastic reaction relative complexity involved in the reset process, here we mainly focus our attention on the investigation of the reset process.

### Chemical reaction model for reset process in Ni:SiO_2_ random resist access memory devices

**Figure 12:**
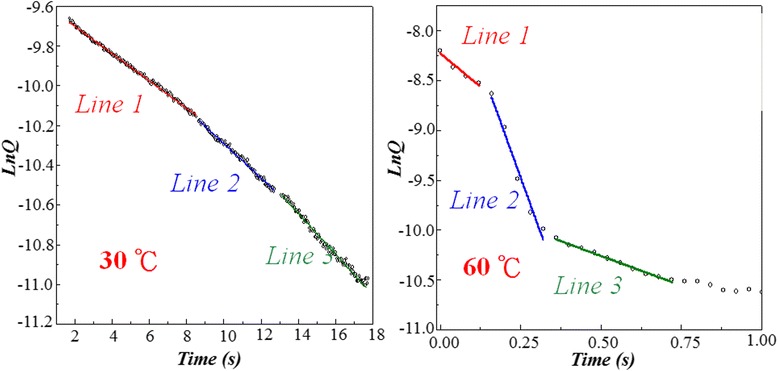
**The Ln(** ***Q*** **)-time curves at 30°C and 60°C calculated from the typical bipolar switching behaviors.**

**Figure 13:**
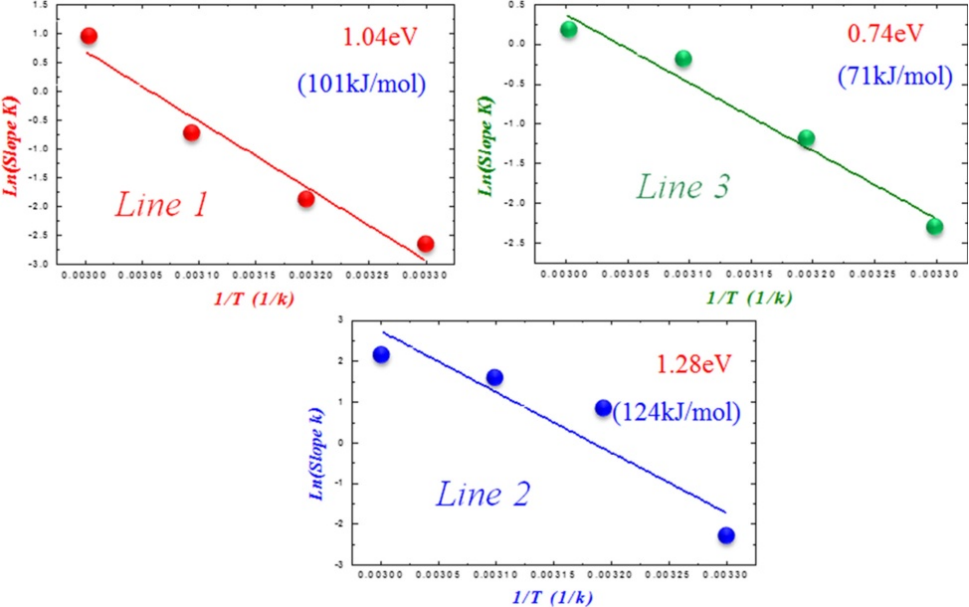
**The Ln(** ***k*** **)-1/T curves with different line segments for Ni:SiO**_**2**_ **RRAM device.**

Figure 12 shows the Ln(*Q*)-time curve at 30°C, from which we found that the curve can be divided into three line segments with unobvious variation on line slope. But when the temperature is increased to 60°C, the Ln(*Q*)-time curve reveals three line segments with different line slope. In order to further investigate the phenomena, the chemical reaction activation energy of different line segments in Ln(*Q*)-time curve was extracted at variable temperature according to the Arrhenius reaction equation, *k* = *A* exp(−*E*_a_/RT), where *k* is reaction rate constant, *A* is reaction intrinsic factor, *E*_a_ is activation energy, and *R* is gas constant. After linear curve fitting and calculation for three line segments in Ln(*k*)-1/T curves as shown in Figure 13, we found that the activation energy of the first line segment, *E*_a1_, is 1.04 eV (101 kJ/mole), the activation energy of the second line, *E*_a2_, is 1.28 eV (124 kJ/mole), and the activation energy of the third segment, *E*_a3_, is 0.7 eV (71 kJ/mole). According to the literature reports [123], *E*_a1_ is close to O-O broken bond energy, *E*_a2_ is close to O-N broken bond energy, and *E*_a3_ is close to the migration activation energy of oxygen ion in silicon oxide solid state material.

**Figure 14:**
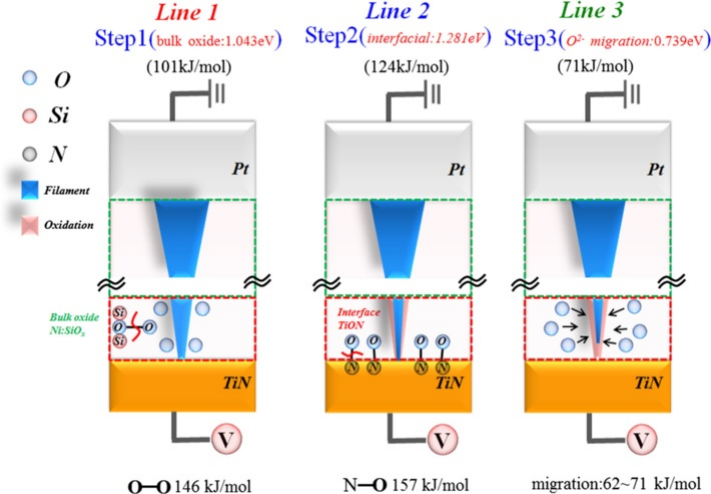
**The schematic diagram of chemical reaction mechanisms in Ni:SiO**_**x**_ **RRAM.** The schematic diagram of chemical reaction mechanisms in Ni:SiO_x_ RRAM to illustrate oxygen ions reaction process during reset process.

According to the results, we propose a model to explain the oxygen ion reaction in Ni:SiO_2_ RRAM device to cause resistance switching process during reset process as shown in Figure 14. Firstly, the oxygen ions would be released due to the O-O bond broken in the bulk of switching layers. Then, the other oxygen ions would be released due to the O-N bond broken in the interface between resistance switching layer and TiN electrode. Finally, the released oxygen ions would be driven by the electric field to the conductive filament in RRAM device, leading to the rupture of filament due to oxidation reaction. Thus, the resistance state of RRAM device will be switched from low-resistance state to high-resistance state to complete the reset process.

## Metal-doped silicon oxide-based resistance random access memory

Although the resistive switching behaviors have been presented in various metal oxide materials [124], silicon-based oxide is a promising material for RRAM applications due to its great compatibility in integrated circuit (IC) processes. Therefore, metal doped into SiO_2_ by cosputtering at room temperature was taken as the resistance switching layer of RRAM in this section. Furthermore, different metal elements were doped into SiO_2_ as the resistance switching layer to investigate the metal dopant type influence on resistive switching behaviors [125-130].

**Figure 15:**
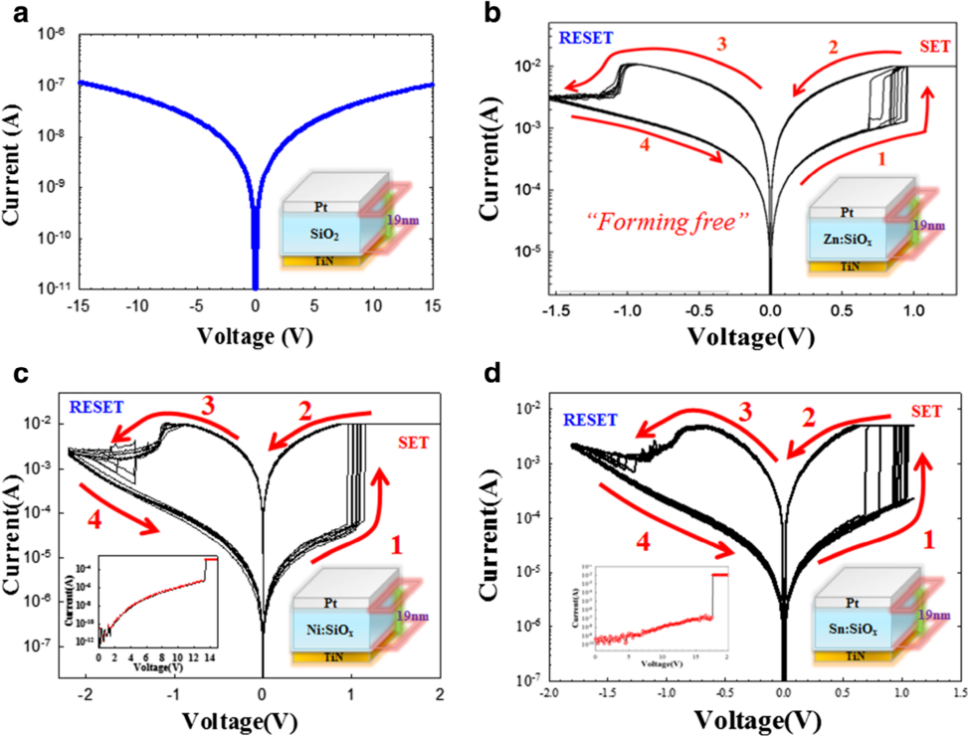
***I-V*** **curve and bipolar curves of Pt/Zn:SiO**_**2**_ **/TiN, Pt/Ni:SiO**_**2**_ **/TiN, and Pt/Sn:SiO**_**2**_ **/TiN devices. (a)** Current–voltage (*I-V*) curve of the Pt/SiO2/TiN sandwich device at room temperature without resistive switching characteristic. Bipolar resistance switching *I-V* curves of **(b)** the Pt/Zn:SiO_2_/TiN device with forming-free property, **(c)** the Pt/Ni:SiO_2_/TiN device, and **(d)** the Pt/Sn:SiO_2_/TiN device.

Figure 15a shows the current-voltage (*I-V*) properties of the control sample with the Pt/SiO_2_/TiN sandwich structure. The sputtered SiO_2_ layer exhibits no reliable RRAM properties even though the applied voltage is biased to a maximum voltage of 15 V. Figure 15b shows bipolar resistance switching characteristics of the Zn:SiO_2_ RRAM device by the DC voltage sweep operations. In particular, the device exhibits resistive switching behavior without forming process. For the Ni:SiO_2_ RRAM devices, the forming process is required to activate the as-deposited samples using DC voltage sweeping with a compliance current. A sudden increase in current occurs at a forming voltage, and the cell was transformed from the initial-resistance state (IRS) to the low-resistance state (LRS). In the Ni:SiO_2_ RRAM device, the resistance ratio of the HRS and the LRS is about 10^3^ times at a reading voltage of 0.1 V, and there is no degradation after continuous *I-V* sweep operations as shown in Figure 15c. In the Sn:SiO*x* RRAM device, the resistance ratio of HRS and LRS is about 10^2^ times at a reading voltage of 0.1 V, which is shown in Figure 15d.

**Figure 16:**
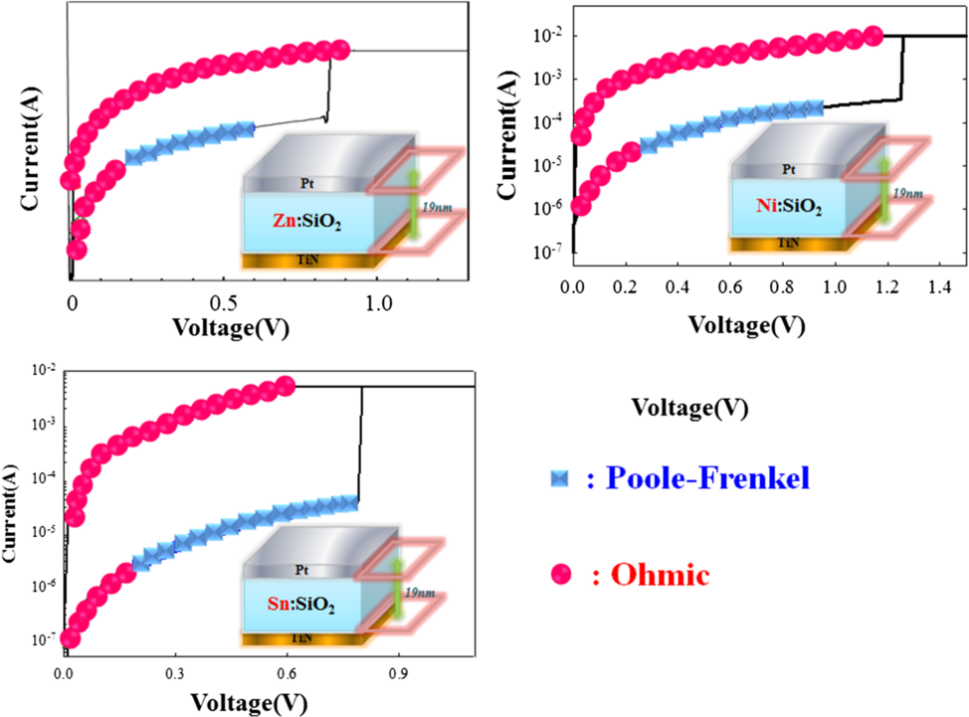
**Electrical characteristics of Pt/metal:SiO**_**2**_ **/TiN memory device.** The current conduction mechanism of HRS is dominated by Poole-Frenkel conduction.

The HRS and LRS of *I-V* curves were analyzed for the current conduction mechanisms in order to further discuss the resistance switching mechanisms in the metal-doped silicon oxide thin film (Zn:SiO_2_, Ni:SiO_2_, Sn:SiO_2_), which is shown in Figure 16. The *I-V* fitting results demonstrated that the current conduction in the HRS in the metal-doped thin film was dominated by the Poole-Frenkel emission mechanism. The Poole-Frenkel conduction is due to thermal emission of trapped electrons into conduction band. Based on the electrical analyses, it could be inferred that metal dopants in SiO_2_ may result in an increased amount of hetero-defects in the film. When the voltage was applied to the film, the electrons were emitted through the hetero-defects, from which we could verify the current conduction dominated by Poole-Frenkel mechanism. On the other hand, the HRS will transform into the LRS when the applied voltage was higher than the set voltage. In this case, results of the current fitting revealed that the current conduction was dominated by Ohmic’s conduction mechanism. We believe that the conductive filament would be formed due to the current flowing through metal-induced defects in the metal-doped silicon oxide film. The conductive filament makes the current conduction mechanism dominated by Ohmic conduction.

**Figure 17:**
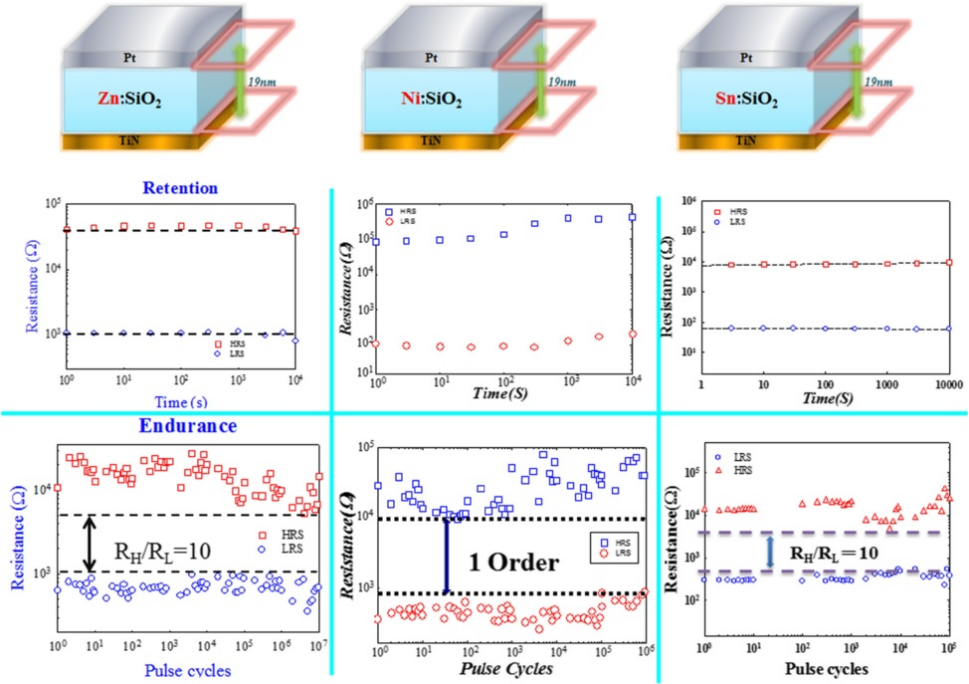
**Retention properties at 85°C and endurance performance of memory device with Pt/metal:SiO**_**2**_ **/TiN structure.** The read voltage was 0.1 V.

In general, endurance and retention tests should be conducted to evaluate the reliability properties of the non-volatile memory devices. Figure 17 shows the retention and endurance characteristics of metal-doped silicon oxide RRAM devices during reliability test. As for the retention performance, the resistance ratio of LRS/HRS remains over one order of magnitude even after 10^4^ s at 85°C. Both HRS and LRS resistance values were measured at 0.1 V. Furthermore, over one-order resistance ratio between the HRS and the LRS can still be obtained even though over 10^5^ cycling bias pulse operations were applied to metal-doped silicon oxide RRAM devices. These endurance and retention tests demonstrate that metal-doped silicon oxide RRAM devices possess acceptable reliabilities.

In order to analyze the influence of metal element on resistance switching characteristics in SiO_2_ thin film, mole fraction of each element in the metal:Si:O film was calculated from the peak areas of XPS spectra, which is shown in Table 1. In addition, endurance and retention testing results are also summarized in Table 1. The results of *I-V* and XPS analyses demonstrate that resistive switching properties for pure SiO_2_ film can be significantly improved by introducing small concentration metal dopants.

**Table 1:** **Comparison of silicon oxide-based RRAM devices with different metal dopants**

	**Zn:SiO**_**2**_	**Ni:SiO**_**2**_	**Sn:SiO**_**2**_
Mole fraction	Zn:Si:O	Ni:Si:O	Sn:Si:O
	4.9:24.9:70.2	2.4:27.9:70.4	0.3:29.5:70.2
Endurance (times)	>10^7^	>10^6^	>10^5^
Retentions at 85°C (sec)	>10^4^	>10^4^	>10^4^

In brief, the reproducible bipolar resistance switching characteristics with acceptable reliability have been successfully achieved by doping few mole fraction of metal into the SiO_2_ film using the cosputtering technique at room temperature. The resistance switching performance of metal-doped SiO_2_ devices are improved due to a localized conduction filament confined in SiO_2_ layer during redox reaction of resistive switching process [125].

## Multilayer graphene oxide-doped oxide-based RRAM

**Figure 18:**
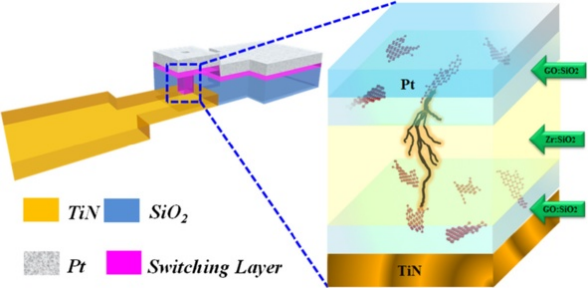
**Schematic device structure of two-sided grapheme oxide RRAM with filament self-aligning growth ability.**

Except for the metal dopants’ influence research on the RRAM working performance, we have also investigated nonmetal-doped SiO*x* characteristics. Here in this section, we present double-ended graphene oxide (GO)-doped silicon oxide-based RRAM with comprehensive excellent quality. What is more, multi-functional property like implementation of complimentary resistive switch (CRS) [131], distinctive multi-bit characteristics in set region, and fine rheostatic behavior in reset process can be achieved. Patterned substrate is fabricated to form 1 × 1 μm size via (shown as Figure 18), in which resistive switching layer is a deposited layer by magnetron cosputtering.

**Figure 19:**
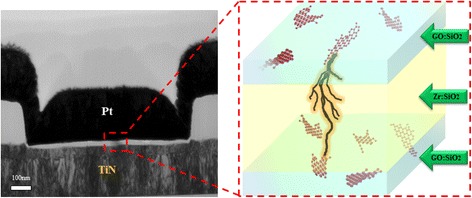
**TEM picture and schematic diagram of filament self-aligning formation process of the triple layer device.** Filament forms from current compliance grapheme oxide (CGO) to switching grapheme oxide (SGO).

Figure 19 is the TEM picture and schematic diagram of filament self-aligning formation process of the triple layer device. During electro-forming process, electrons tend to conduct through the current compliance graphene oxide (CGO) flake with lowest resistance and trigger the accumulation of metal precipitates to form a filament. The filament grows in a directional way, approaching to the switching graphene oxide (SGO) flake to form a current leakage path.

**Figure 20:**
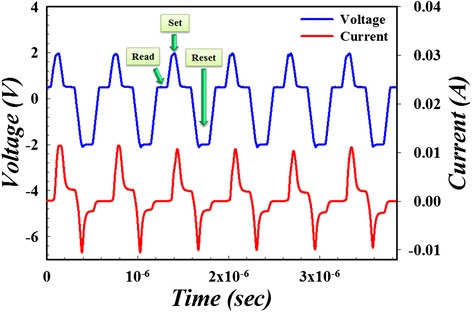
**Uniform resistive switching with current self-limiting property of double-ended graphene oxide RRAM under AC test.**

The double-ended GO triple switching layer structure possesses instinctive ability to restrict multi-filament growth that may lead to multi-switching points. The multi-filament formation reflects on the electrical characteristics as instable current-voltage curve, especially in the reset process and HRS [67]. Moreover, it is notable to mention the current compliance graphene oxide flake in top GO:SiO_2_ layer. Due to its finite size which defines the maximal flux of electrons, current is restricted automatically, making it easier to avoid the filament over-formation and excessive rupture. Also, it is quite normal for single layer RRAM, especially for the metal or metal oxide-based RRAM, to exhibit unstable HRS and over-shooting phenomenon in LRS, owing to the stochastic rupture of metal filament and excessive accumulation of metal precipitates, respectively [67,132]. Furthermore, the more frequent the filament over formation/rupture happens, the more unstable the current-voltage curve within, thus making it much more possible for RRAM device breakdown and degrading in continuous write/erase process. Therefore, by combining the current self-limiting property and uniform conductive path formation ability, the two-sided GO structure RRAM can undoubtedly achieve the goal of uniform resistive switching and this is confirmed by AC pulse switch test shown in Figure 20. From the testing result, the switching process of the two-sided GO structure device shows outstanding uniformity and stability even in the conventional rough unstable reset region.

**Figure 21:**
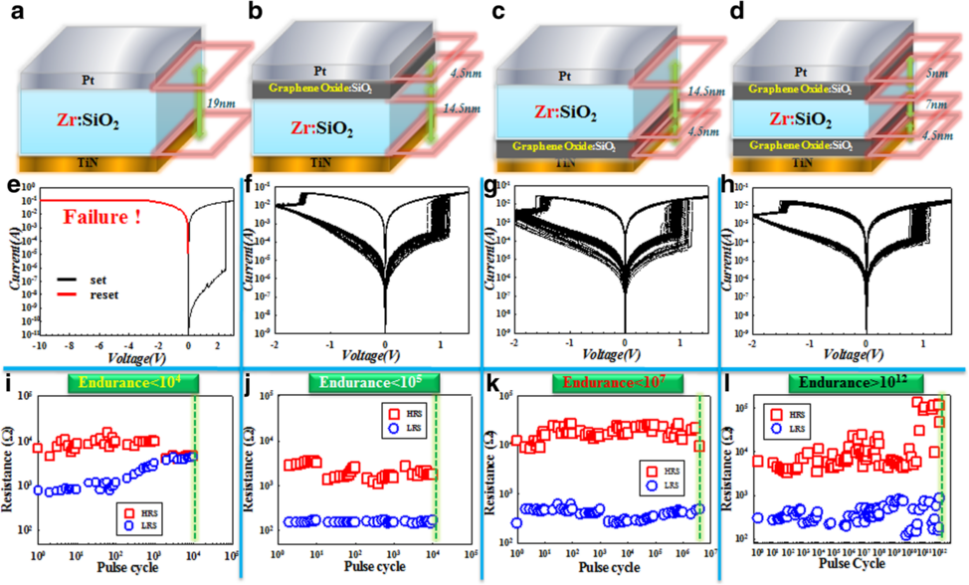
**Switching layer structure, 100-cycle current-voltage sweep, and corresponding endurance property test. a** to **d** are the switching layer structures of the four kinds of devices. **e** to **h** are the 100-cycle current-voltage sweep without equipment current compliance. Insets of the figures are the corresponding HRS and LRS distribution with a reading voltage of 0.1 V. **i** to **l** are the endurance performance of each device.

To verify the excellent electrical property of the two-sided GO RRAM device, four kinds of devices are fabricated and tested, which are shown in Figure 21. In Figure 21a,b,c, and d are the schematic device structures, Figure 21e to Figure 21l are their corresponding DC (without equipment current compliance) and AC endurance test results. From DC testing result, we can find that single Zr:SiO_2_ exhibits no current self-limiting property but GO:SiO_2_/Zr:SiO_2_, Zr:SiO_2_/GO:SiO_2_, and GO:SiO_2_/Zr:SiO_2_/GO:SiO_2_ structure devices own intrinsic current restriction ability. It can be also observed that the two-sided GO RRAM device shows uniform switching during 100-cycle switching, and the memory window also remains stable compared with one-sided GO RRAM device (insets of Figure 21e-h are the corresponding distribution of HRS and LRS read at 0.1 V). To the AC endurance test, the two-sided GO device can achieve up to more than 10^12^ cycles. It is the first time for silicon oxide-based RRAM to reach this endurance value, comparable to the previous result of TaO-based RRAM [132].

**Figure 22:**
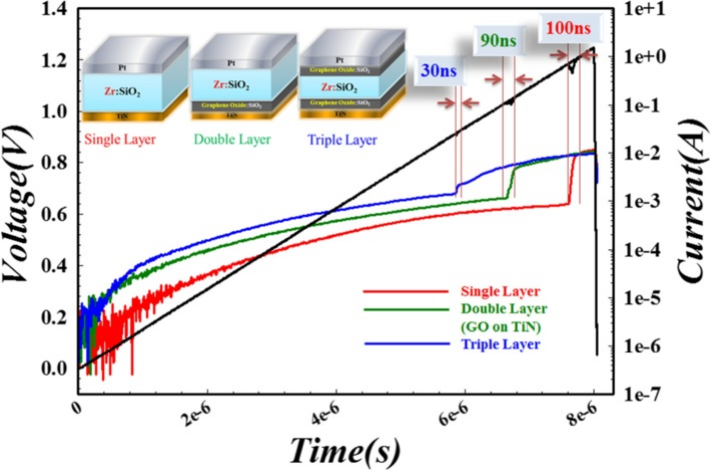
**Set process response time testing.** The devices are applied with a triangle voltage pulse. The resistance sharp transition time for triple switching layer, double (Pt/Zr:SiO_2_/C:SiO_2_/TiN) switching layer, and single switching layer devices are around 30, 90, and 100 ns, respectively.

**Figure 23:**
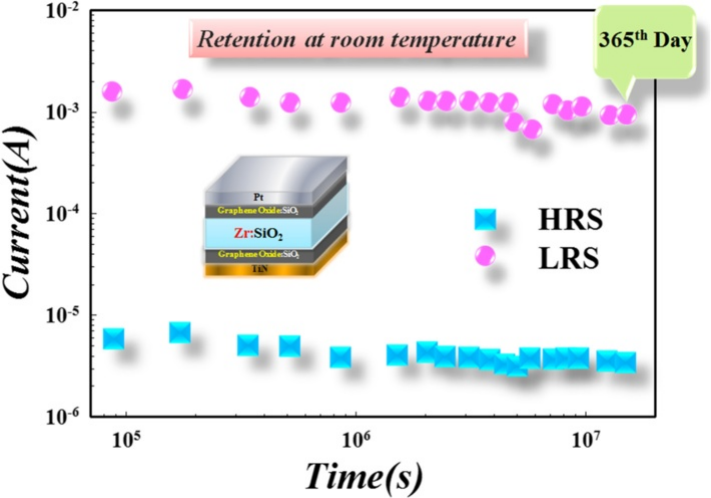
**Retention properties of two-sided GO layer devices.** The retention at room temperature can be achieved over 365th days with no degradation.

**Figure 24:**
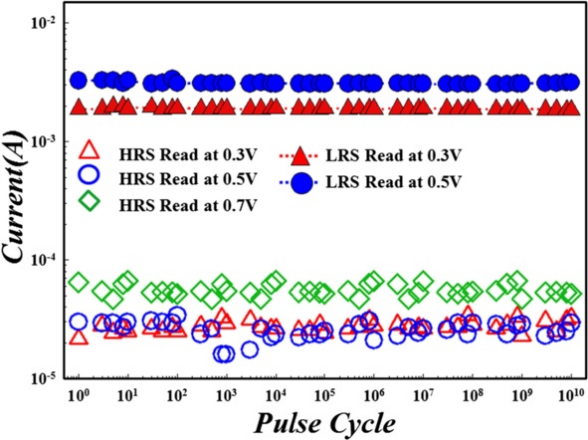
**Read disturbance immunity of two-sided GO RRAM.** To the LRS and the HRS, it can achieve up to −0.5 V and 0.7 V 10^10^-cycle continuous read pulse for reading test without resistance degradation, respectively.

In order to further confirm the reliability and performance of two-sided GO device, set response time, retention properties, and read disturbance immunity are tested, which are shown in Figures 22, 23, and 24.

**Figure 25:**
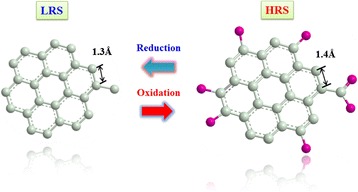
**Oxidation simulation result of GO.** With the addition of oxygen-contained chemical groups, carbon-carbon bonds are stretched.

**Figure 26:**
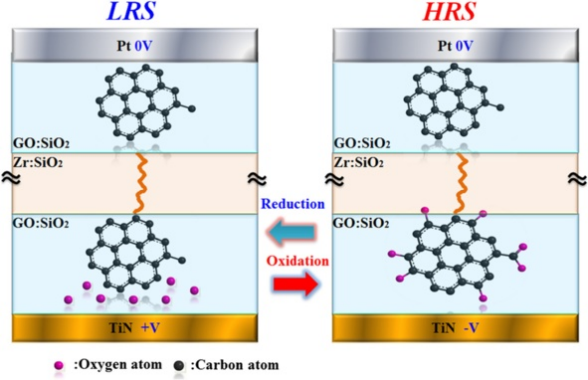
**Current conduction model of double-ended GO structure RRAM.**

To the two-sided GO device, resistive switching happens in the bottom GO:SiO_2_ layer, which results from the oxygen-contained chemical group adsorption and desorption. With the addition of more oxygen-contained chemical groups to GO, its resistance increases and meanwhile the hopping distance for electrons becomes longer. This is confirmed by ChemBio simulation (see Figure 25) and also previous research results from our group and other groups [6,133]. The current conduction model is shown schematically in Figure 26. Both GO flakes in the top and bottom GO:SiO_2_ layer work as the current limiter, but the bottom GO flake also plays a dominant role in the resistive switching. With the help of metal filament to concentrate electrical filed, oxygen-contained chemical groups can be attracted to or repelled from GO.

**Figure 27:**
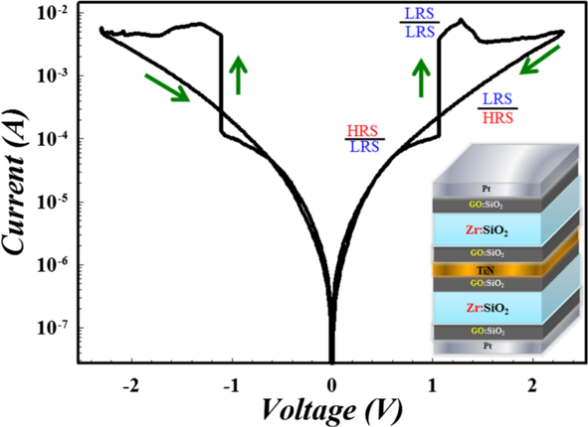
**Complementary resistive switching with a two-sided GO RRAM stacking structure.**

**Figure 28:**
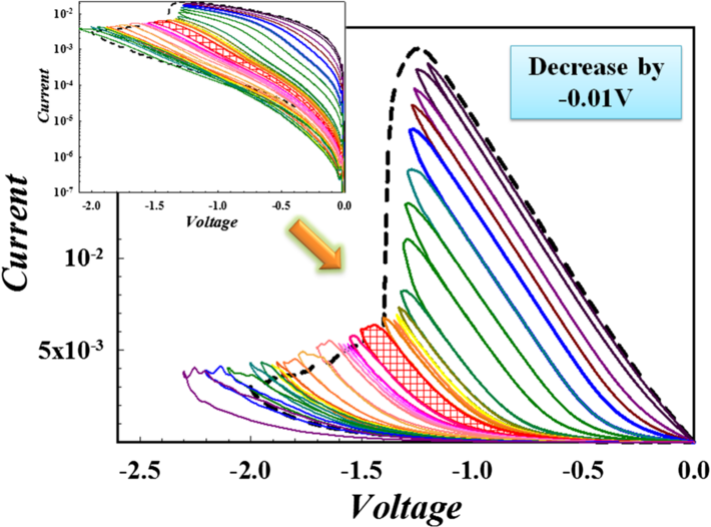
**Fine multi-bit property in reset process for the two-sided GO RRAM.**

As it is the ultimate aim for device to be integrated, the scalability for RRAM device is an important factor. Owing to the inborn sneak current for cross-bar architecture, CRS memory is a promising candidate for the mass production of RRAM for its simple structure and elimination of any selector element [82]. Together with our device-instinctive current self-limiting ability, we successfully fabricated CRS memory, which is shown in Figure 27. Furthermore, in order to improve the density of integration, multi-bit storage is a simple and characteristic method for RRAM device, which needs no consideration for complex downscaling or multi-stacking. By decreasing only 0.1 V for the two-sided GO device, we obtain fine multi-bit property in reset region (see Figure 28) and this is also the first time for RRAM device to achieve such fineness.

**Figure 29:**
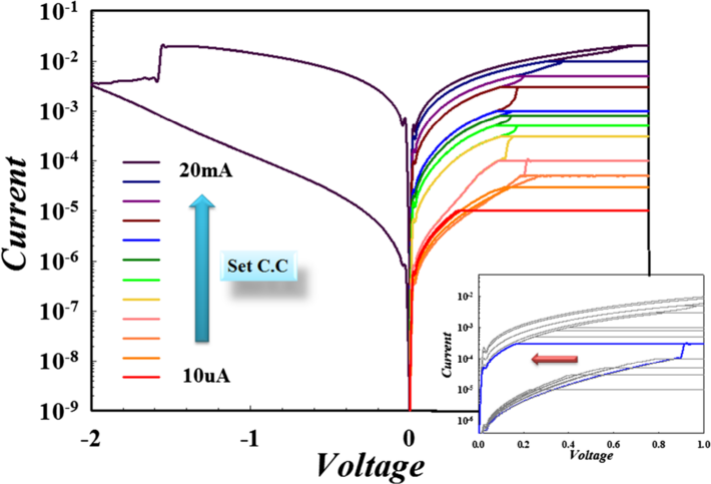
**Multi-bit property in set process by varying set current compliance.**

What is more, multi-bit can be also achieved by increasing current compliance in set region (see Figure 29) and this is quite different from the single set process of Zr:SiO_2_ structure device (see the ole sweeping cycle multi-bit property; the double-ended GO-doped silicon oxide-based structure is a promising technique to the future mass production of RRAM for its comprehensive preferable working properties (bottom right inset of Figure 29) or metal-based RRAM device.

In summary, with its strong intrinsic current self-limiting ability, CRS implementation capability, and whole sweeping cycle multi-bit property, the double-ended GO-doped silicon oxide-based structure is a promising technique to the future mass production of RRAM for its comprehensive preferable working properties.

## New low temperature process technology: supercritical fluids

**Figure 30:**
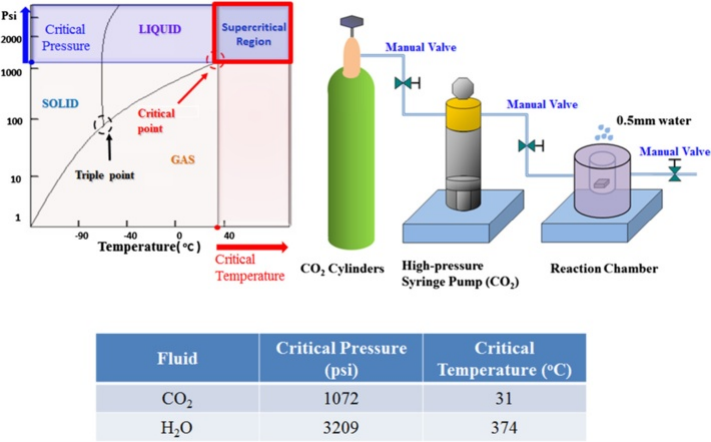
**Physical properties and schematic diagram of supercritical CO**_**2**_ **fluids systems.**

The supercritical CO_2_ (SCCO_2_) fluid technology was used to improve the dielectric properties and performance of various thin film transistors (TFTs), such as hydrogenated amorphous-silicon TFTs and ZnO TFTs [134-138]. Therefore, the effect of SCCO_2_ treatment on RRAM was worthy to evaluate for low temperature process [139-146]. Figure 30 shows the critical point definition for supercritical fluid and the schematic diagram for SCCO_2_ treatment facilities. Supercritical phase is distinctive with its characteristics of high penetration of gas and solubility of liquid. The supercritical water fluid has tremendous oxidation property [147]. However, high critical temperature and high critical pressure are essential conditions to achieve supercritical water fluid. So it is difficult to realize through modern facilities (the table of Figure 30). Thus we adopted CO_2_, as it is easier for CO_2_ to achieve supercritical point and meanwhile it is relatively inert, which will not react with the treated materials. By adding a little water into supercritical CO_2_ fluids, the liquid water can approach to the supercritical fluid phase due to the mixed SCCO_2_ fluids close to idea solution.

In this section, we choose tin metal-doped silicon oxide (Sn:SiO*x*) as the resistance switching layer of RRAM to discuss the reaction mechanism of SCCO_2_ fluid on Sn:SiO*x* RRAM to explain the reason of electrical property improvement.

**Figure 31:**
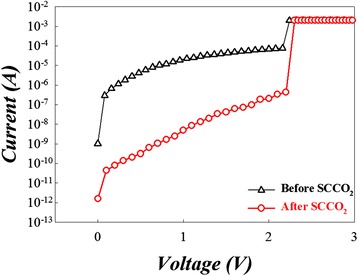
**The forming** ***I-V*** **curves of the Sn:SiO**_**x**_ **RRAM devices before and after SCCO**_**2**_ **treatment [** 146 **].**

From Figure 31, we can find the leakage current of the Sn:SiO*x* RRAM devices after SCCO_2_ treatment was lower than that of pre-treatment devices. However, the forming voltage of RRAM after SCCO_2_ treatment is not changed. This phenomenon has been reported by our previous study [135].

**Figure 32:**
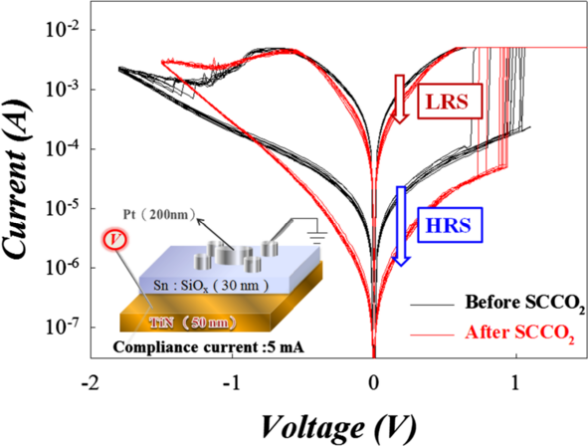
**The** ***I-V*** **curves are the resistive switching characteristics of Sn:SiO**_**x**_ **film [** 146 **].**

The voltage sweep bias was applied on TiN electrode with the grounded Pt electrode as shown in the bottom left inset of Figure 32. The electrical current-voltage properties of the Sn:SiO*x* devices were compared before and after SCCO_2_ treatment (Figure 32). We can find the working current of Sn:SiO*x* devices is remarkably reduced especially in the HRS after SCCO_2_ treatment.

**Figure 33:**
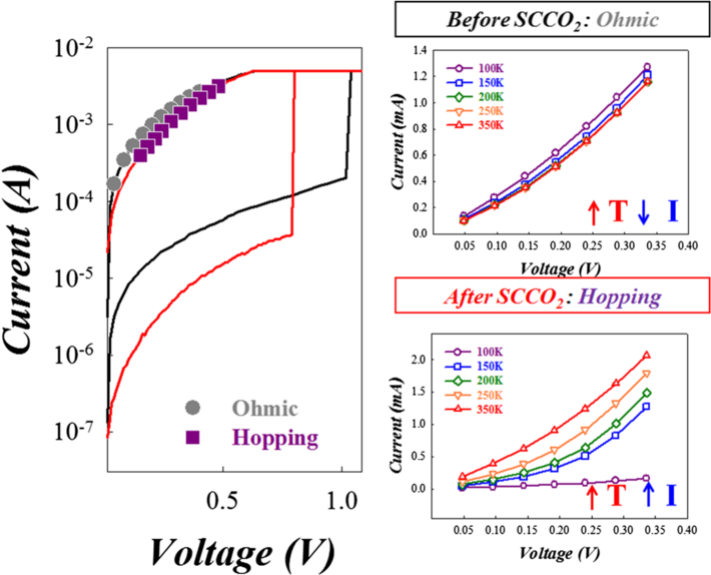
**The** ***I-V*** **curves in the LRS of Sn:SiO**_**x**_ **devices before and after SCCO**_**2**_ **treatment.** The *I-V* diagrams on the right were obtained through vary-temperature measurement before and after SCCO_2_ treatment, respectively [146].

To investigate the reason of reduction on working current of Sn:SiO*x*, we analyzed the current conduction mechanism of Sn:SiO*x* thin film with and without SCCO_2_ treatment as shown in Figure 33. The carrier transport in LRS state of Sn:SiO*x* device was dominated by Ohmic conduction in the Sn:SiO*x* layer. After SCCO_2_ treatment, the current conduction mechanism will transfer to hopping conduction because of the change of material properties.

**Figure 34:**
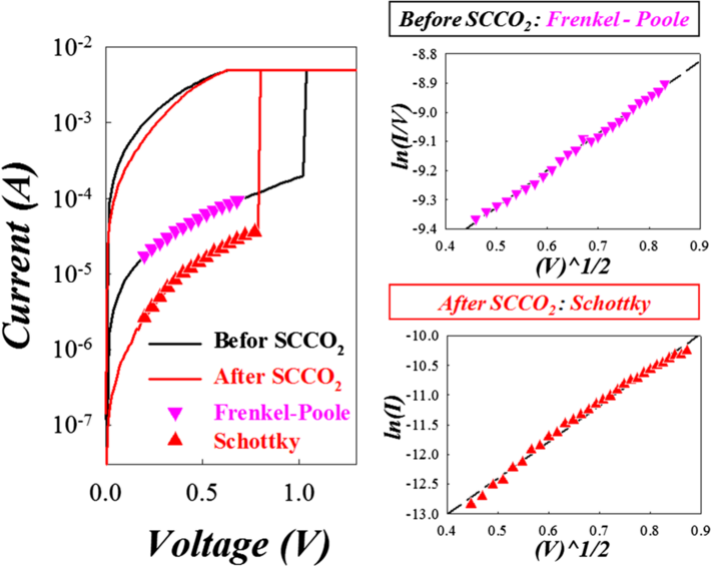
**The** ***I-V*** **curves in the HRS of Sn:SiO**_**x**_ **devices before and after SCCO**_**2**_ **treatment.** The fitting results of *I-V* curves in the HRS for the devices before and after SCCO_2_ treatment shown as the right side [146].

In addition, we also analyzed the current conduction mechanism in HRS of Sn:SiO*x* with and without SCCO_2_ treatment as shown in Figure 34. The relationship in the curve of ln(*I*/*V*) versus the square root of the applied voltage (*V*^1/2^) is linear. The results revealed that the carrier transport of Sn:SiO*x* film was dominated by Poole-Frenkel conduction due to the trap in the film. After SCCO_2_ treatment, the current conduction mechanism will transfer to Schottky emission because of the improvement of dielectric properties.

**Figure 35:**
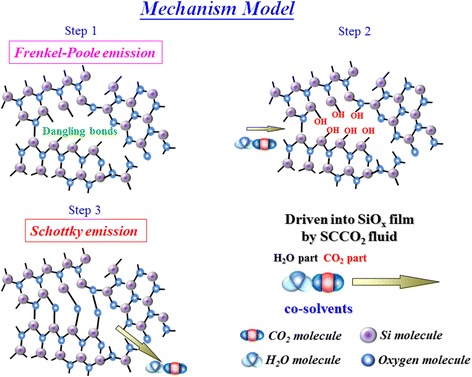
**The schematic diagram of hydration-dehydration reaction mechanism on Sn:SiO**_**x**_ **film** **[** 146 **].**

In order to explain the current reduction of SCCO_2_ treatment mechanism clearly, we proposed a reaction mode to explain the characteristics of the SCCO_2_-treated Sn:SiO*x* film, as shown in Figure 35. As the sample was put into the water-mixed SCCO_2_ fluid environment, the H_2_O molecule was carried into the grain boundary of Sn:SiO*x* film by SCCO_2_ fluid, which is attributed to the high penetration ability of SCCO_2_ fluid. Then, dehydration of neighbor hydroxyl groups was induced in supercritical fluids so as to form Si-O-Si and Sn-O-Si network-like bonding in the film, which is called as hydration-dehydration reaction of SCCO_2_ fluids in Sn:SiO*x* film.

**Figure 36:**
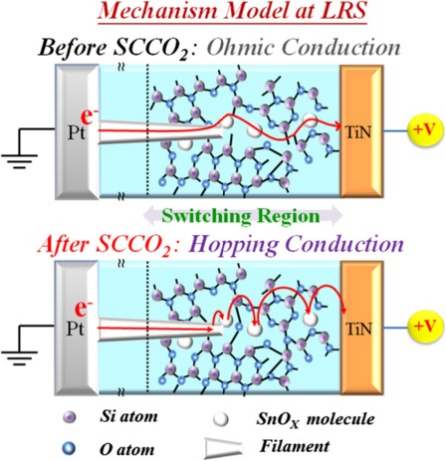
**The schematic diagram of carrier hopping model in Sn:SiO**_**x**_ **film after SCCO**_**2**_ **treatment** **[** 146 **].**

As for the LRS of Sn:SiO*x* film, the conductive filament will be formed in Sn:SiO*x* film after the forming process. Then, the carriers were transported through these dangling bonds, leading to the current conduction dominated by Ohmic conduction. If the Sn:SiO*x* film was put into the SCCO_2_ fluid environment, the tin metal in Sn:SiO*x* thin film will be isolated due to hydration-dehydration reaction by SCCO_2_ treatment. Only if the conductive filament was applied to the Sn:SiO*x* film, the tin metal will be isolated through SCCO_2_ treatment, leading to the electrical current conduction in LRS of Sn:SiO*x* film transferred to hopping conduction as shown in Figure 36.

**Figure 37:**
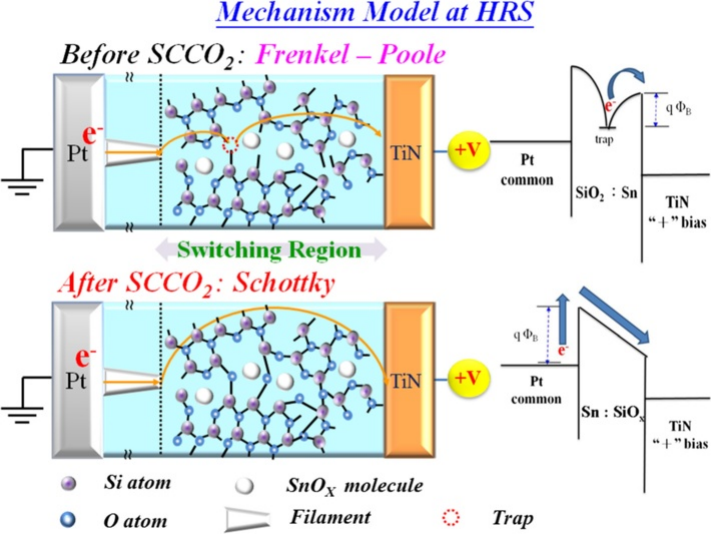
**The schematic diagram of the transfer on carrier conduction mechanism in Sn:SiO**_**x**_ **film after SCCO**_**2**_ **treatment** **[** 146 **].**

On the other hand, the electrical current conduction in HRS of Sn:SiO_*x*_ film will be transferred to Schottky emission from Poole-Frenkel conduction through passivation effect of SCCO_2_ as shown in Figure 37. This implicates that the SCCO_2_ can improve dielectric properties of the switching film, leading to the reduction of the working current and the power consumption of RRAM devices.

To sum up, the operation current of metal-doped silicon oxide RRAM device can be reduced by SCCO_2_ fluid treatment. According to the analysis of resistive switching physical mechanism, water molecules can be brought into the film to passivate the dangling bonds in the resistive switching layer due to hydration-dehydration reaction, and SCCO_2_ fluid treatment is undoubtedly a kind of attractive technology for the RRAM device working performance improvement. In order to discuss the advantages and disadvantages of the overall performances of silicon oxide-based RRAM devices by different fabricated methods, the comparison of silicon oxide-based RRAM devices with different fabricated methods is shown in Table 2. If the metal-doped silicon oxide is adopted as the resistive switching layer of RRAM device, the reliability performance including endurance property, retention, and operation stability can conform to the requirement of non-volatile memory. Furthermore, the outstanding performance of oxide-based RRAM (endurance property >10^12^ cycles, self-current limiting operation) can be demonstrated with multilayer GO-doped silicon oxide layer structure. Finally, we can reduce the operation current of silicon oxide-based RRAM for portable electronic product applications by SCCO_2_ fluid treatment.

**Table 2:** **Comparison of silicon oxide based RRAM devices with different fabricated methods**

	**Metal-doped SiO**_**2**_	**Multilayer GO-doped SiO**_**2**_	**SCCO**_**2**_ **treatment**
Operation current	Typical	Typical	Low
Operation stability	Good	Good	Good
Endurance (times)	>10^5^	>10^12^	>10^5^
Retention at 85°C (sec)	>10^4^	>10^4^	>10^4^

## Conclusions and outlook

In conclusion, we have reported the bipolar resistance switching characteristics and physical mechanisms of the oxide-based RRAM devices through ultra-cryogenic and fast *I-V* measurement systems. The experimental results show that the setting voltage is related to setting time, with *ΔV*_set_ inversely proportional to (*Δt*_set_)^− 0.5^. These results can be used to correctly allocate voltage and time to control RRAM working characteristics. In addition, the switching mechanism is proved to be the redox reaction through a specific constant switching energy. The dynamic switching mechanisms during reset procedure in RRAM were also clarified by a sequential experimental design. According to the electrical measurement and analysis in ultra-cryogenic measurement systems, we verify that the ultra-fast switching speed of RRAM is attributed to several atomic reaction procedures in virtue of the atomic-level quantized phenomena in ultra-cryogenic temperature situation. Based on the investigation of resistance switching physical mechanism in RRAM devices, we designed a high performance multi-functional RRAM device by modifying the resistance switching layers, new device structures, and novel process technology. The reproducible bipolar resistance switching characteristics with acceptable reliability have been successfully achieved by doping few mole fraction of metal into the SiO_2_ film using the cosputtering technique at room temperature. With its strong intrinsic current self-limiting ability, CRS implementation capability, and whole sweeping cycle multi-bit property, the double-ended GO-doped silicon oxide-based structure is a promising technique to the future mass production of RRAM. Furthermore, the operation current of metal-doped silicon oxide RRAM device can be reduced by SCCO_2_ fluid treatment. Based on the analysis of resistive switching physical mechanism, the water molecules can be brought into the film to passivate the dangling bonds in the resistive switching layer due to hydration-dehydration reactions. In the future, we hope to spend more efforts to build up a universal model on resistive switching behaviors of oxide-based RRAM so as to boost the mass production of RRAM and also facilitate the fabrication of three-dimensional stacking structure storage circuits.
